# Virulence Characteristics and Antibiotic Resistance Profiles of Shiga Toxin-Producing *Escherichia coli* Isolates from Diverse Sources

**DOI:** 10.3390/antibiotics9090587

**Published:** 2020-09-08

**Authors:** Momna Rubab, Deog-Hwan Oh

**Affiliations:** Department of Food Science and Biotechnology, College of Agriculture and Life Sciences, Kangwon National University, Chuncheon 200-701, Korea; rubab.momna@kangwon.ac.kr

**Keywords:** Shiga toxin-producing *Escherichia coli*, virulence, human, PCR, antimicrobial resistance

## Abstract

Shiga toxin-producing *Escherichia coli* (STEC) is an enteric pathogen that causes several gastrointestinal ailments in humans across the world. STEC’s ability to cause ailment is attributed to the presence of a broad range of known and putative virulence factors (VFs) including those that encode Shiga toxins. A total of 51 *E. coli* strains belonging to serogroups O26, O45, O103, O104, O113, O121, O145, and O157 were tested for the presence of nine VFs via PCR and for their susceptibility to 17 frequently used antibiotics using the disc diffusion method. The isolates belonged to eight different serotypes, including eight O serogroups and 12 H types. The frequency of the presence of key VFs were *stx1* (76.47%), *stx2* (86.27%), *eae* (100%), *ehxA* (98.03%), *nleA* (100%), *ureC* (94.11%), *iha* (96.07%), *subA* (9.80%), and *saa* (94.11%) in the *E. coli* strains. All *E. coli* strains carried seven or more distinct VFs and, among these, four isolates harbored all tested VFs. In addition, all *E. coli* strains had a high degree of antibiotic resistance and were multidrug resistant (MDR). These results show a high incidence frequency of VFs and heterogeneity of VFs and MDR profiles of *E. coli* strains. Moreover, half of the *E. coli* isolates (74.5%) were resistant to > 9 classes of antibiotics (more than 50% of the tested antibiotics). Thus, our findings highlight the importance of appropriate epidemiological and microbiological surveillance and control measures to prevent STEC disease in humans worldwide.

## 1. Introduction

*Escherichia coli* is ubiquitous in nature, often found in soil, water, food, human, and animal intestinal tracts. However, *E. coli* can also act as a pathogen in a wide range of conditions, from enteric diseases to extraintestinal infections. *E. coli* strains which cause enteric diseases or diarrhea are known as diarrheagenic *E. coli* (DEC) and are divided into six distinct pathotypes based on clinical, epidemiological, and molecular criteria [1]. Among them, enteropathogenic *E. coli* (EPEC) is the predominant cause of diarrhea in developing countries [1,2,3,4,5] and enterohemorrhagic *E. coli* (EHEC) is attributed to the foodborne outbreaks in developed countries and can cause bloody diarrhea due to the production of Shiga toxins (*stx*s) and is known as Shiga toxin-producing *E. coli* (STEC). Among the DEC, STEC includes the most dangerous strains and more than 400 serotypes that produce *stxs* have been identified, and this term was created since *E. coli* species possess the toxin, which is more or less identical to that produced by *Shigella dysentery* type I [6]. STEC possesses a broad range of virulence factors (VFs), which are encoded by chromosomal genes, and they are often located in pathogenicity islands (PAIs) or plasmids, with the production of *stxs* being the most crucial resulting in endothelial cell damage and possible hemolytic uremic syndrome (HUS) [7,8]. In addition, STEC is responsible for 2.8 million illnesses, and 3890 cases per year of enteric disease in humans, globally [8]. Shiga toxin is classified into two types, Shiga toxin type 1 (*stx1*) and Shiga toxin type 2 *(stx2*) and subtypes which dominate the pathogenicity of STEC, and it is known to be an important factor in differentiating the severity of illness [2,9,10], but cannot be solely responsible for full pathogenicity, as a vast arsenal of VFs are essential for STEC pathogenicity. Thus, STEC possesses other VFs necessary for infection, such as intimin (*eae*), which is an outer membrane encoded by the locus for enterocyte effacement (LEE) and is essential for the intimate adherence of *eae*-positive STEC strains to the host’s intestine and, eventually, for the attaching and effacing (A/E) lesions frequently perceived in STEC infections. In addition, several STEC strains contain a number of plasmid-encoded VFs, including enterohemolysin (pO157; *ehxA*). The toxin *ehxA* is a heat-labile pore-forming toxin that causes hemolysis of host red blood cells and the possession of *ehxA* by a STEC strain has been attributed to HUS [11]. Other plasmid-encoded (pO113) VFs include a subtilase cytotoxin gene (*subA*; triggers apoptosis in human cells) and an autoagglutinating adhesin gene (*saa*; associated with the absence of LEE) [12] also recognized as key plasmid-encoded VFs. The gene *subA* suppresses the host’s immune system and allows STEC to adhere to enterocytes. Growing evidence suggests that differences in virulence between pathogenic and nonpathogenic bacterial strains can be attributed to VFs in pathogenicity islands [13]. Several PAIs of STEC, including genes encoding OI-43/48, OI-57, and OI-71, are absent in nonpathogenic *E. coli* and are viewed as STEC VFs. These genes have been used in molecular risk assessment research to classify STEC serotypes into various seropathotypes depending on whether a specific serotype in humans has been involved in mild, severe ailment, or no disease at all. Furthermore, numerous genes located on OI-43/48, including *IrgA* homolog adhesin (encodes an adhesin; *iha*) and *ureC* (encode urease resistance), and on OI-71, including *nleA* (effector; disrupts protein secretion) are considered appropriate virulence markers in STEC serotypes involved in severe human ailments and outbreaks but their individual role in the pathogenesis of human infection is still poorly understood [2]. In addition, these genes are mainly found in high risk (HUS) STEC strains and are associated with colonization and survival in the host, and may interfere with signaling pathways during inflammation [14].

In the last decade, the STEC has gained substantial attention as a global public health concern for various sporadic infections and major outbreaks due to the emergence of resistance to multiple antibiotics as there are fewer, or often no, effective antibiotics available for infections caused by such bacteria. Furthermore, multidrug resistance (MDR) in *E. coli* strains has increased in recent years, causing serious problems in healthcare settings worldwide [1,15,16]. MDR represents one of the notable global health challenges of this century and the global increase in its spread is a major issue, particularly in developing countries where there is limited control over the quality, distribution, and use of antibiotics in human medicine, veterinary medicine, and food animal farming [17]. MDR bacterial infections have increased at an alarming rate due to the tremendous dissemination of antibiotic resistance determinants [18,19,20] and MDR pathogens and infections thereof are expected to cause 10 million deaths per year by 2050 [21,22]. Even though the role of commensal bacteria in providing antibiotic resistance has long been recognized, however, these bacteria have not been extensively studied [18]. *E. coli* comprises only a small fraction of the human gastrointestinal tract’s bacterial flora, but it is not a significant reservoir. Resistance in commensal *E. coli* from healthy patients was first demonstrated over 50 years ago, and several recent studies have demonstrated the high or increased incidence of MDR in commensal *E. coli* from healthy children and adults from various countries [23,24,25]. Currently, the use of antibiotics to treat STEC infection in humans is controversial and not recommended in many countries according to the current clinical guidelines, as some antibiotics can induce the production of Shiga toxin [8,26,27]. However, antibiotic resistance is a matter of growing concern due to the wide spread of *E. coli* resistance to all antibiotics used in human therapy, and the dispersion of resistance via mobile genetic elements. Additionally, STEC bacteriophages may carry antibiotic resistance-encoding genes that can be transferred to naïve *E. coli* which are then transformed into antibiotic-resistant strains [8,21].

Hence, bacteria with VFs and antibiotic resistance should be carefully monitored. Currently, there is limited information available on the prevalence of antibiotic resistance in *E. coli* isolates from humans and food samples. Understanding the prevalence of resistance to antibiotics, especially for critically important antibiotics, in *E. coli* isolates from human and food samples, will provide the useful information for developing risk management options to mitigate the spread of resistance. This research was performed to investigate the diversity and distribution of the major virulence-associated factors (*stx1*, *stx2*, *iha*, *ureC*, *eae*, *uidA*, *nleA*, *ehxA*, *subA*, and *saa*) in previously isolated strains from diverse sources including foods, animal carcasses and feces, and humans by using PCR. The second objective was to determine the strains’ potential as human pathogens by employing 17 frequently used antibiotics in clinical practices. This study emphasizes on the importance of preventing the spread of *E. coli* isolates that harbor both antibiotic resistance and virulence genes and the overall objective of this research was to contribute to STEC surveillance and gain insight into STEC strains’ molecular epidemiology in human diseases.

## 2. Materials and Methods

### 2.1. Bacterial Strains and Growth Conditions

A total of 51 *E. coli* strains were investigated, obtained from the US Food Fermentation Laboratory Culture Collection (USDA ARS, Raleigh, N.C., USA) and the USDA ARS Eastern Regional Research Center (Wyndmoor, Pa., USA). The strains were derived from humans (*n* = 40), domestic animals (*n* = 9), and food (*n* = 2). The *E. coli* and non-targeted reference strains that were used in this study are described in detail in Table 1. All strains were routinely propagated at 37 °C in Luria–Bertani (LB) broth (Difco, Becton, Dicknison, MD, USA) under aerobic conditions.

### 2.2. DNA Extraction

The genomic DNA was extracted using a GeneAll® Exgene^TM^ Cell SV genomic DNA purification kit (GeneAll^TM^, Seoul, Republic of Korea) according to the manufacturer’s instructions from 1 mL overnight culture of STEC strains grown at 37 °C. The concentrations of extracted DNA were determined using Eppendorf Biospectrometer® fluorescence (Eppendorf, Hamburg, Germany) at 260 nm. The genomic DNA was stored at −20 °C until use.

### 2.3. Detection of Virulence Genes

All 51 isolates were screened for genetic markers of virulence associated with STEC by conventional polymerase chain reaction (PCR; Mygenie32 Thermal Block, Bioneer Co., Daejeon, Korea) using primers and conditions described previously for targeting bacteriophage-encoded Shiga toxin genes (*stx1*, *stx2*), an attaching and effacing gene (*eae*), putative adhesin genes (*saa*, *iha*), a toxicity gene (*subA*), and a plasmid-encoded virulence factor (*ehxA*). The strains were also characterized by the use of O71 (MRA; based on the presence of various O-island nle genes) associated with pathogenicity molecular risk assessment. The primers for the detection of targeted genes have been described previously and are listed in Table 2. The *uidA* marker was use to confirm that all were *E. coli*. The *E. coli* strain ATCC 35150 served as a positive control for STEC strains and K-12 (ATCC 700926; MG1655) served as a negative control for the nine VFs investigated in this work. The PCR assay was carried out using Accupower Taq PCR PreMix (Bioneer Co., Daejeon, Korea) in a total volume of 20 µL comprising 2 µL of DNA template. Amplified PCR products were loaded into single wells of 2% agarose gel containing SafeView^TM^ (ABM, Richmond, BC, Canada). After subsequent electrophoresis using an electrophoresis system (Mupid-exU, Mupid, Tokyo, Japan) at 100 V for 30 min in 1× Tris-acetate-EDTA (TAE) buffer, the gel was visualized using a UV transilluminator (Gel Doc 2000; Bio-Rad, Hercules, CA, USA), where a 100-bp DNA ladder (ThermoFisher Scientific, Seoul, Korea) was used in each agarose run and used as a molecular weight marker.

### 2.4. Antibiotic Susceptibility Testing

The antibiotic susceptibility of the *E. coli* strains as per the guidelines of the Clinical and Laboratory Standards Institute (CLSI) were examined using a disc diffusion method [35]. The following 17 antibiotics were tested: ampicillin, methicillin, penicillin, vancomycin, erythromycin, clindamycin, ciprofloxacin, chloramphenicol, gentamicin, imipenem, meropenem, streptomycin, tetracycline, kanamycin, nalidixic acid, novobiocin, and tigecycline. *E. coli* ATCC 25922, which is sensitive to all the drugs, was used as the control strain [6,36,37,38,39]. The results were used to classify the strains as resistant or susceptible to a specific antibiotic using standard reference values recommended by the CLSI National Committee [35]. The strains were classified as MDR when they presented resistance to ≥ 1 antibiotic in 3 ≥ antibiotic categories [40].

## 3. Results

### 3.1. Virulence Gene Profile of STEC Strains from Diverse Resources 

**Figure 1:**
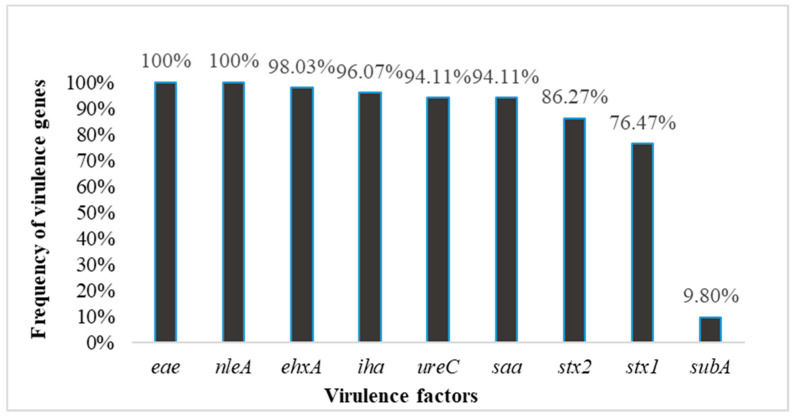
Virulence-associated factor profiles of STEC isolates from diverse sources.

The isolated strains belonged to eight different serotypes, including eight O serogroups and 12 H types: 13.7% belonged to each serotype except O121 (12%) and O157 (5.88%) (Table 1). The strains expressed 11 different H antigens: H- (3.92%), H2 (15.6%), H4 (5.88%), H6 (1.96%), H7 (7.84%), H8 (5.88%), H11 (17.6%), H12 (3.92%), H19 (3.92%), H21 (1.92%), H25 (3.92%), and H-nonmotile (NM; 27.4%). The *E. coli* strains analyzed in this study were isolated from various sources, with the majority isolated from humans (Table 1). The presence of nine toxin-encoding genes was investigated using PCR in all *E. coli* strains. PCR revealed that all strains tested positive for *eae*, *nleA*, and *uidA* genes. The detection rate of *stx1* and *stx2* was 76.47 and 86.27%, respectively. The prevalences of the plasmid-encoded genes were as follows: *iha* (96.07%), *saa* (94.11%), *ehxA* (98.03%), *subA* (9.80%), and *ureC* (94.11%) (Figure 1). Our findings revealed that most of the *E. coli* isolates contained multiple and heterogeneous VFs and different gene combinations were observed in our investigation, as recorded in Table 3.

### 3.2. Antibiotic Resistance of STEC Strains

The resistance profiles of the *E. coli* strains against the tested antibiotics are described in Table 4. All antibiotic susceptibility results were interpreted using the breakpoints of the CLSI guidelines. Among the 17 tested antibiotics, resistance was most frequent for gentamicin (50/51, 98.03%) and novobiocin (49/51, 96.07%), followed by kanamycin (39/51, 76.47%), streptomycin (42/51, 82.35%), ampicillin (37/51, 72.54%), and tetracycline (22/51, 43.13%). Meanwhile, all the strains were resistant against methicillin, penicillin, vancomycin, and erythromycin. Additionally, all *E. coli* strains were susceptible to imipenem and meropenem. The high prevalence of antibiotic susceptibility was detected for nalidixic acid (50/51, 98.0%), followed by chloramphenicol (43/51, 84.3%), tetracycline (19/51, 37.25%), ciprofloxacin (17/51, 33.33%), clindamycin (15/51, 29.41%), and tigecycline (13/51, 25.49%). Moreover, half of the *E. coli* isolated (38/51, 74.5%) were resistant to > 9 classes of antibiotics (more than 50% of the tested antibiotics) (Table 5).

### 3.3. Frequency of Virulence Factor Occurrence in Isolated E. coli Strains Exhibiting Antibiotic Resistance

The frequencies of VF occurrence in isolated *E. coli* strains exhibiting antibiotic resistance are detailed in Table 6. The frequencies for *eae*, *ehxA*, and *nleA* among the resistant *E. coli* isolates were nearly > 98%, whereas those of *ureC* and *saa* were > 95%. Moreover, the frequencies of *iha*, *stx2*, *stx1*, and *subA* in the resistant isolates were higher than 90, 86, 80, and 9%, respectively.

## 4. Discussion

The concept of molecular risk assessment [41] has been used effectively to classify STEC strains into those that are attributed to outbreaks and life-threatening diseases in humans and those causing less severe disease or that are not involved in human disease. *E. coli* strains have been characterized by serotype and the presence and subtype of VFs, as well as other toxins and plasmid-associated adherence and virulence factors. Thus, in this study, we investigated the presence of VFs in *E. coli* strains to broaden the knowledge of the properties of *E. coli* strains isolated from diverse sources. Recent epidemiological studies have revealed that the STEC serotypes O26, O103, O111, O145, and O157 are highly related to human infections (they may account for up to 80% of human STEC infections) [8] and our results are in agreement with reports from other countries describing the high pathogenic potential of strains associated with these serogroups [2]. The presence of non-LEE effector genes encoded by O-island O1-71 is highly attributed to strains that were often involved in outbreaks and serious disease in humans [41,42,43]. We observed that *stx2*-harboring isolates were more frequent than *stx1*-harboring isolates. The dominant combinations of VFs present in the strains studied were *stx1*, *stx2*, and *eae* (76.4% of strains). The distribution pattern of VFs was similar to the STEC strains isolated from domestic animals in Mexico [44]. In France, the eminent toxin genotype was that of *stx2*-carrying STEC strains. From these findings, it seems that STEC strains carrying the *stx1* gene are more often confronted than those carrying *stx2*. Possession of OI-43/48, OI-71, and non-LEE effectors genes together with *stx2*, *eae* and a whole plasmid is the hallmark of highly virulent STEC strains that are frequently associated with outbreaks and serious diseases such as hemorrhagic colitis (HC) and HUS [8]. Since the carriage of combinations of *stx* genes has been correlated with severity of the disease, the *stx* gene profile provides us an overview of the pathogenic potential of these STEC strains from diverse sources. STEC strains that carry both *stx2* and the *eae* genes were more often associated with severe disease. Interestingly, all of the isolates were harboring the *eae* gene, and it is reported that a significant majority of human STEC isolates obtained from HC or HUS patients contained *eae* [45]. The presence of *subA* in the *E. coli* strains was similar to that observed in some STEC strains isolated from human infections in the USA and Australia [46]. It was previously reported that the amount of *stx2* production is capable of determining the severity of diseases caused by STEC strains. Results of this characterization have identified that *E. coli* strains, defined by the presence of *eae*, *subA*, and the *nleA* genes, may be considered a significant food safety threat. Plasmid-encoded VFs enhance pathogenesis and contribute to the survival of STEC in humans [47], however, the pathogenic mechanisms of STEC infection are only partially understood. The varying prevalence of various VFs indicates that STEC strains are heterogeneous and it has been hypothesized that the combination of these genes may complement the Shiga toxin effect and enhance its virulence among STEC strains. The majority of strains in our research were positive for key VFs. Most of the strains carried the full complement of OI-43/48 VFs and all non-LEE-encoding effector genes. There is no specific pattern of virulence markers capable of interfering with the pathogenic potential of a given STEC isolate, and the search for a broad set of VFs has become the best strategy for measuring the microbiological and clinical risks that these pathogens may pose.

The 17 most frequently used antibiotics in clinical practice were employed to assess the actual frequency of antibiotic resistance in 51 *E. coli* strains. Generally, antibiotics are divided mainly into three categories on the basis of their functions (Table 4). While MDR was seen, however, there was no common pattern of resistance. It is important to note that all strains were resistant to at least three different classes of antibiotic agents and were considered as MDR. The majority of the *E. coli* strains of our research showed a high prevalence of resistance against first-line antibiotics (commonly prescribed oral antibiotics) such as ampicillin, penicillin, methicillin, gentamicin, vancomycin, novobiocin, streptomycin, kanamycin, and erythromycin (Table 4). The similar results of antibiotic resistance of *E. coli* strains were reported in developing countries such as Brazil, Turkey, China, and Ghana [1,4,5,45]. Our data exhibit a high resistance rate in *E. coli* strains that is comparable to those reported in previous studies. These results illustrate the growing extent of the misuse of antibiotics in clinical practices. In particular, *E. coli* strain resistance to methicillin, penicillin, vancomycin, and novobiocin reached 100%. The resistance rates of these *E. coli* strains were higher than reported in developing countries [3,48]. These kinds of MDR STEC strains pose a serious threat to human health by affecting treatment against them. It was previously reported that patients infected with STEC should not be treated with antibiotics due to the risk of developing HUS [26,49]. *E. coli* forms part of the human gut’s commensal flora and has been identified as the predominant reservoir of genes for antibiotic resistance [20]. These resistance genes are stable once acquired and are easily transferable to pathogenic bacteria [50]. These transfers have effectively changed the etiological and pathogenic character of bacterial species [51]. The majority of the STEC serotypes found in this study have also been reported in other countries. Depending on comparison by serotype and sequence type with human strains and the prevalence of VFs, the STEC strains could have a higher potential to cause human disease. Documented data showed that STEC strains isolated from human and food samples show a high prevalence of resistance to various types of antibiotics, including aminoglycosides, tetracycline, penicillin, and chloramphenicol. Molecular epidemiological studies have shown that the presence of certain antibiotic resistance genes, including the genes that encode resistance against tetracycline (*tetA* and *tetB*), ampicillin (*CITM*), gentamicin (*aac (3)-IV*), chloramphenicol (*cat1* and *cmlA*), and aminoglycosides (*aadA1*), is the key cause of antibiotic resistance in STEC [52,53]. With regard to macrolide antibiotics (erythromycin), *E. coli* is an enteric bacterium, which are often non-susceptible due to the presence of chromosomal efflux pumps (*mel*) or cellular impermeability [54,55]. A number of different mechanisms have been reported for the macrolide resistance of Gram-negative bacteria. These mechanisms include the presence of a number of genes, such as two ester genes (*ere*(A) and *ere*(B)) [56,57,58], phosphorylase genes (*mph*(A), *mph*(B), and *mph*(D)) [59], and one rRNA methylase gene (*erm*(B)) [56,60,61]. In addition, if they acquire macrolide resistance genes, such as *mef*(A) and *mef*(B), that may increase their resistance levels further [49]. We found that the pattern of phenotypic resistance of STEC strains was supported by the genotypic resistance of STEC strains isolated from various samples followed by a high prevalence of antibiotic resistance genes [52]. Several studies have recorded that the prevalence of antibiotic-resistant *E. coli* has increased since 1950 [62]. An alarming increase in the prevalence of MDR *E. coli* strains all over the world has been reported and this is a result of the spread of plasmids and other genetic elements. This has made antibiotic resistance a major public health issue globally [62]. *E. coli* is an important food safety and public health concern because of its pathogenicity and potential for MDR.

Lastly, but most importantly, we found that *E. coli* strains harbor a high level of VFs in addition to high MDR (Table 3 and Table 4). These results explain how *E. coli* strains can effectively invade the human body and evade antibiotic treatment. The results of this research demonstrate that MDR *E. coli* strains harbor a high frequency of VFs and their VF profiles are highly heterogeneous. These results suggest that use of antibiotics needs to be monitored by the private, public, and agricultural sectors as certain antibiotics can induce the production of *stx* and thus encourage the onset of severe disease symptoms in humans. The improper use of antibiotics has become a public health problem worldwide in healthcare settings. Given the importance of *E. coli* in food safety and public health, our findings on the prevalence of antibiotic resistance and VFs provide valuable information for risk management strategies to protect public health. The monitoring of the antibiotic resistance of STEC is pivotal due to the likelihood of the horizontal transfer of resistance genes from notorious STEC strains to other pathogens. In addition, the monitoring process will help in developing new treatment approaches and help in establishing effective control strategies that assist in stopping the spread of resistance. Hence, the molecular typing and contentious monitoring of antibiotic resistance could be helpful in developing efficacious control strategies against STEC and in formulating new antibiotics with reduced tendency for antibiotic resistance. The diversity in the prevalence of *stx* genes, enterotoxin genes, and other virulence-related genes in this study and the other studies can be attributed to the geographical origin of samples, the sample size, the handling of the collected samples, the number of strains examined, the type of the examined VFs, and the role of the examined VFs in the pathogenesis of the disease. Surveillance data suggest that resistance in *E. coli* is consistently highest for antibiotics that have been in use in human and veterinary medicine for the longest period of time [62]. The past two decades have witnessed major increases in the emergence and spread of MDR bacteria and increasing resistance to newer compounds, such as fluoroquinolones and certain cephalosporins [62]. Thus, surveillance and control measures need to be intensified to prevent further spread of these strains in the world.

In summary, *E. coli* is a significant cause of diarrheal and foodborne outbreaks, resulting in severe economic losses. This research demonstrates a high prevalence and heterogeneity of VF profiles among human MDR *E. coli* strains. The virulotyping revealed that the majority of *E. coli* strains were positive for *stx1*, *stx2*, *eae*, *ehxA*, *ureC*, *nleA*, and *iha* but *subA* was observed in a very small number of isolates. The serotype O26 strains possess the highest number of virulence-associated factors. The majority of isolates were resistant to two or more antibiotics that are commonly used in clinical medicine for the treatment of various bacterial diseases. However, all the 51 isolates were sensitive to imipenem and meropenem and, therefore, these drugs could be the drugs of choice in the treatment of STEC infections. We conclude that appropriate efforts should be focused on surveillance and that control measures to prevent/reduce further the spread of such microorganisms are crucial. However, further research using whole genome sequences would therefore be required to better understand the prevalence of VFs and antibiotic resistance in *E. coli* strains that may arise in this important human pathogen. The continuous monitoring and screening for MDR foodborne pathogens should be performed. Taken together, this knowledge will provide a better understanding of the risks associated with STEC and will aid in the development of appropriate and tailored intervention strategies.

## Figures and Tables

**Table 1:** List of Shiga toxin-producing *E. coli* isolates analyzed in this study.

ID No.	Serotype	Source
B444	O26:H11	H
B445	O26:H11	H
B446	O26:H11	H
B447	O26:H11	H
B448	O26:H11	H
B449	O26:H11	H
B450	O26:H11	H
B451	O45:NM	Co (calf)
B452	O45:H2	D
B453	O45:H2	H
B454	O45:H2	Go
B455	O45:H2	R
B456	O45:H12	Co
B457	O45:H2	Co (calf)
B458	O103:H2	H
B459	O103:H25	H
B460	O103:H25	H
B461	O103:H2	H
B462	O103:H11	H
B463	O103:H6	H
B464	O103:H11	H
B465	O104:H4	H
B466	O104:H4	H
B467	O104:H21	H
B468	O104:H7	Ca
B469	O104:H4	H
B470	O104:H2	Co (feces)
B471	O104:H12	Co
B472	O111:H-	H
B473	O111:NM	H
B474	O111:NM	H
B475	O111:NM	H
B476	O111:H8	H
B477	O111:H8	H
B478	O111:H8	H
B479	O121:NM	H
B480	O121:H19	H
B481	O121:NM	H
B482	O121:H19	H
B483	O121:NM	H
B484	O121:NM	H
B485	O145:NM	H
B486	O145:NM	H
B487	O145:H-	H
B488	O145:NM	H
B489	O145:NM	Co
B490	O145:NM	H
B491	O145:NM	H
B492	O157:H7	H
B493	O157:H7	H
B494	O157:H7	Gb

**Table 2:** List of DNA oligonucleotides used in this study for PCR amplification.

Target Gene	Primer	Nucleotide Sequence (5′—3′)	PCR Conditions	Amplicon Size (bp)	Reference
*stx*1	*stx*1-F	GAAAGCGATGCAGCTATTA	95 °C for 15 min, 95 °C for 30 s, 60 °C for 40 s, 72 °C for 30 s, 40 cycles, 72 °C for 3 min	789	[28]
	*stx*1-R	GGATAATTTGTTTGCAGTTG			
*stx*2	*stx*2-F	TATTATTTAAATGGGTACTGTGC		1073	
	*stx*2-R	ATGTGTCATCCTCATTATACTTG			
*eae*	*eae*-F	CATTGATCAGGATTTTTCTGGTGATA		102	[29]
	*eae*-R	CTCATGCGGAAATAGCCGTTA			
*ehx*A	*ehx*A-F	CGTTAAGGAACAGGAGGTGTCAGTA		142	
	*ehx*A-R	ATCATGTTTTCCGCCAATGAG			
*subA*	*subA*-F	TATGGCTTCCCTCATTGCC	94 °C for 5 min, 94 °C for 45 s, 50 °C for 30 s, 72 °C for 30 s, 30 cycles, 72 °C for 3 min	556	[30]
	*subA*-R	TATAGCTGTTGCTTCTGACG			
*Saa*	*saa*-F	CGTGATGAACAGGCTATTGC	94 °C for 5 min, 94 °C for 45 s, 56 °C for 30 s, 72 °C for 30 s, 30 cycles, 72 °C for 7 min	119	[31]
	*saa*-R	ATGGACATGCCTGTGGCAAC			
*nle*A	*nle*A-F	ATGAACATTCAACCGACCATAC	94 °C for 5 min, 94 °C for 30 s, 55 °C for 60 s, 35 cycles, 72 °C for 2.5 min	1296	[32]
	*nle*A-R	GACTCTTGTTTCTTGGATTATATCAAA			
*ureC*	*ureC*-F	TCTAACGCCACAACCTGTAC	94 °C for 3 min, 94 °C for 60 s, 60 °C for 60 s, 72 °C for 60 s, 35 cycles, 72 °C for 2.5 min	397	[33]
	*ureC*-R	GAGGAAGGCAGAATATTGGG			
*iha*	*iha*-F	CAGTTCAGTTTCGCATTCACC	95 °C for 15 min, 94 °C for 30 s, 55 °C for 60 s, 72 °C for 60 s, 30 cycles, 72 °C for 5 min	1305	[34]
	*iha*-R	GTATGGCTCTGATGCGATG			

**Table 3:** Pattern of distribution of essential chromosomal and plasmid-encoded virulence-associated factors in STEC isolates from diverse sources.

No. of Virulence Genes	Virulence Gene Profile	No. (%) of Bacterial Strains	Total No. (%)
6 genes	*stx1*, *stx2*, *eae*, *ehxA*, *saa*, *nleA*	2 (3.92)	4 (7.84)
	*stx1*, *stx2*, *eae*, *nleA*, *iha*, *ureC*	1 (1.96)	
	*eae*, *nleA*, *ehxA*, *saa*, *iha*, *ureC*	1 (1.96)	
7 genes	*stx1*, *stx2*, *eae*, *ehxA*, *saa*, *iha*, *nleA*	1 (1.96)	19 (37.25)
	*stx1*, *eae*, *ehxA*, *saa*, *iha*, *nleA*, *ureC*	6 (11.76)	
	*stx2*, *eae*, *ehxA*, *saa*, *iha*, *nleA*, *ureC*	10 (19.60)	
	*stx1*, *stx2*, *eae*, *ehxA*, *iha*, *nleA*, *ureC*	2 (3.92)	
8 genes	*stx1*, *stx2*, *eae*, *ehxA*, *saa*, *iha*, *nleA*, *ureC*	23 (45.09)	24 (47.05)
	*stx2*, *eae*, *ehxA*, *saa*, *iha*, *nleA*, *ureC*, *subA*	1 (1.96)	
9 genes	*stx1*, *stx2*, *eae*, *ehxA*, *iha*, *nleA*, *ureC*, *subA*	4 (7.84)	4 (7.84)

**Table 4:** Multidrug resistance patterns of STEC isolates from diverse sources.

Antibiotic	Resistant n (%)	Intermediate n (%)	Susceptible n (%)
**Bacterial protein synthesis**			
Gentamicin	50 (98.03)	0 (0)	1 (1.96)
Kanamycin	39 (76.47)	5 (9.8)	7 (13.72)
Streptomycin	42 (82.35)	7 (13.72)	2 (3.92)
Erythromycin	51 (100)	0 (0)	0 (0)
Tetracycline	22 (43.13)	10 (19.6)	19 (37.25)
Clindamycin	17 (33.33)	19 (37.25)	15 (29.41)
Tigecycline	7 (13.72)	31 (60.78)	13 (25.49)
Chloramphenicol	6 (11.76)	2 (3.92)	43 (84.31)
**Cell wall synthesis**			
Ampicillin	37 (72.54)	12 (23.52)	2 (3.92)
Penicillin	51 (100)	0 (0)	0 (0)
Methicillin	51 (100)	0 (0)	0 (0)
Vancomycin	51 (100)	0 (0)	0 (0)
Imipenem	0 (0)	0 (0)	51 (100)
Meropenem	0 (0)	0 (0)	51 (100)
**Nucleic acid targeting**			
Novobiocin	49 (96.07)	2 (3.92)	0 (0)
*Inhibit DNA synthesis*			
Ciprofloxacin	17 (33.33)	17 (33.33)	17 (33.33)
Nalidixic acid	1 (1.96)	0 (0)	50 (98.03)

**Table 5:** Number of STEC isolates resistant to different classes of antibiotics.

Different Classes of Antibiotics		6	7	8	9	10	11	12
**Isolates**	N	2	1	10	11	10	9	7
	%	3.92	1.96	19.6	21.5	19.6	17.6	13.7

**Table 6:** Frequency of virulence factors among antibiotic-resistant STEC isolates from diverse sources.

Antibiotics (n)	Virulence Factors, n (%)								
	*stx1*	*stx2*	*eae*	*ehxA*	*nleA*	*iha*	*saa*	*ureC*	*subA*
Gentamicin (50)	38 (76.00)	43 (86.00)	49 (98.00)	49 (98.00)	49 (98.00)	49 (98.00)	49 (98.00)	49 (98.00)	5 (10.00)
Kanamycin (39)	31 (79.48)	33 (84.61)	39 (100)	39 (100)	39 (100)	38 (97.43)	38 (97.43)	37 (94.87)	5 (12.82)
Streptomycin (42)	33 (78.57)	35 (83.33)	42 (100)	42 (100)	42 (100)	40 (95.23)	41 (97.61)	39 (92.85)	5 (11.90)
Erythromycin (51)	39 (76.47)	44 (86.27)	51 (100)	50 (98.03)	51 (100)	49 (96.07)	48 (94.11)	48 (94.11)	5 (9.80)
Tetracycline (22)	18 (81.81)	17 (77.27)	22 (100)	22 (100)	22 (100)	22 (100)	22 (100)	22 (100)	3 (1.36)
Clindamycin (17)	15 (88.23)	15 (88.23)	17 (100)	17 (100)	17 (100)	17 (100)	16 (94.11)	17 (100)	2 (11.76)
Tigecycline (7)	6 (85.71)	6 (85.71)	7 (100)	7 (100)	7 (100)	7 (100)	7 (100)	7 (100)	1 (14.28)
Ampicillin (37)	27 (72.97)	34 (91.89)	37 (100)	36 (97.29)	37 (100)	36 (97.29)	34 (91.89)	35 (94.59)	2 (5.40)
Penicillin (51)	39 (76.47)	44 (86.27)	51 (100)	50 (98.03)	51 (100)	49 (96.07)	48 (94.11)	48 (94.11)	5 (9.80)
Methicillin (51)	39 (76.47)	44 (86.27)	51 (100)	50 (98.03)	51 (100)	49 (96.07)	48 (94.11)	48 (94.11)	5 (9.80)
Vancomycin (51)	39 (76.47)	44 (86.27)	51 (100)	50 (98.03)	51 (100)	49 (96.07)	48 (94.11)	48 (94.11)	5 (9.80)
Novobiocin (49)	38 (77.55)	43 (87.75)	49 (100)	48 (97.95)	49 (100)	47 (95.91)	46 (93.87)	46 (93.87)	5 (10.20)
Chloramphenicol (6)	5 (83.33)	6 (100)	6 (100)	6 (100)	6 (100)	6 (100)	6 (100)	6 (100)	1 (16.66)
Ciprofloxacin (17)	13 (76.47)	14 (82.35)	17 (100)	16 (94.11)	17 (100)	16 (94.11)	15 (88.23)	16 (94.11)	2 (11.76)
Nalidixic acid (1)	1 (100)	1 (100)	1 (100)	1 (100)	1 (100)	1 (100)	1 (100)	1 (100)	0 (0)

## Abbreviations

STEC	Shiga toxin-producing *Escherichia coli*
VFs	Virulence factors
PCR	Polymerase chain reaction
MDR	Multidrug resistance
DEC	Diarrheagenic *E. coli*
EPEC	Enteropathogenic *E. coli*
EHEC	Enterohemorrhagic *E. coli*
HUS	Hemolytic uremic syndrome
LEE	Locus for enterocyte effacement
CLSI	Clinical and Laboratory Standards Institute
